# Association between unhygienic menstrual management practices and prevalence of lower reproductive tract infections: a hospital-based cross-sectional study in Odisha, India

**DOI:** 10.1186/s12879-018-3384-2

**Published:** 2018-09-21

**Authors:** Belen Torondel, Shalini Sinha, Jyoti Ranjan Mohanty, Tapoja Swain, Pranati Sahoo, Bijaya Panda, Arati Nayak, Mary Bara, Bibiana Bilung, Oliver Cumming, Pinaki Panigrahi, Padmalaya Das

**Affiliations:** 1 Department of Disease Control, London School of Hygiene and Tropical Medicine, London, UK; 2 School of Life Sciences, AIPH University, Bhubaneswar, Odisha India; 3 Department of Obstetrics and Gynaecology, Capital Hospital, Bhubaneswar, Odisha India; 4 Department of Obstetrics and Gynaecology, Ispat General Hospital, Rourkela, Odisha India; 5 Department of Epidemiology and Pediatrics, Center for Global Health and Development, college of Public Health, University of Nebraska Medical Center, Omaha, USA

**Keywords:** Menstrual hygiene management, Bacterial vaginosis, Candidiasis, *Trichomonas vaginalis*, Reproductive tract infections

## Abstract

**Background:**

The extent to which reproductive tract infections (RTIs) are associated with poor menstrual hygiene management (MHM) practices has not been extensively studied. We aimed to determine whether poor menstrual hygiene practices were associated with three common infections of the lower reproductive tract; Bacterial vaginosis (BV), Candida, and *Trichomonas vaginalis* (TV).

**Methods:**

Non-pregnant women of reproductive age (18–45 years) and attending one of two hospitals in Odisha, India, between April 2015 and February 2016 were recruited for the study. A standardized questionnaire was used to collect information on: MHM practices, clinical symptoms for the three infections, and socio-economic and demographic information. Specimens from posterior vaginal fornix were collected using swabs for diagnosis of BV, Candida and TV infection.

**Results:**

A total of 558 women were recruited for the study of whom 62.4% were diagnosed with at least one of the three tested infections and 52% presented with one or more RTI symptoms. BV was the most prevalent infection (41%), followed by Candida infection (34%) and TV infection (5.6%). After adjustment for potentially confounding factors, women diagnosed with Candida infection were more likely to use reusable absorbent material (aPRR = 1.54, 95%CI 1.2–2.0) and practice lower frequency of personal washing (aPRR = 1.34, 95%CI 1.07–1.7). Women with BV were more likely to practice personal washing less frequently (aPRR = 1.25, 95%CI 1.0–1.5), change absorbent material outside a toilet facility (aPRR = 1.21, 95%CI 1.0–1.48) whilst a higher frequency of absorbent material changing was protective (aPRR = 0.56, 95%CI 0.4–0.75). No studied factors were found to be associated with TV infection. In addition, among women reusing absorbent material, Candida but not BV or TV - infection was more frequent who dried their pads inside their houses and who stored the cloth hidden in the toilet compartment.

**Conclusion:**

The results of our study add to growing number of studies which demonstrate a strong and consistent association between poor menstrual hygiene practices and higher prevalence of lower RTIs.

**Electronic supplementary material:**

The online version of this article (10.1186/s12879-018-3384-2) contains supplementary material, which is available to authorized users.

## Introduction

Menstrual hygiene management (MHM) practices vary by socio-cultural context, educational background and economic status, and there is wide variation in MHM practices between and within countries [1, 2]. MHM is characterized by practices such as the type of absorbent material used and the frequency changed, associated body washing, the methods of washing, drying and storing reusable pads as well as other contextual factors, such as the location of menstruation-related changing and washing practices. These practices can be influenced by water, sanitation and hygiene (WASH) facilities at the household level, and the quality of, and access to, these facilities varies significantly between and within countries [3]. MHM practices can be unhygienic and inconvenient, particularly in resource-constrained settings, with poor WASH access [4], and have been found to be associated with different reproductive tract infections {BV, and vulvo-vaginal candidiasis (VVC)} [4] and with psychosocial stress outcomes [5].

Reproductive Tract Infections (RTIs) are a major public health concern worldwide and are particularly common in low-income settings [6, 7]. The prevalence of RTIs and sexually transmitted infection (STIs) in women aged 15–44 years in Odisha was 35.2% in 2002–2004 [8]. Another study from the state of West Bengal, India, has reported that girls with higher socioeconomic status generally had both safer MHM practices and fewer gynecological problems [9]. The most common lower reproductive tract infections are bacterial vaginosis (BV), vulvo-vaginal candidiasis (VVC), and *Trichomonas vaginalis* (TV) [10]. BV, the most common RTI, is characterized by an alteration in the vaginal microbiome with a decline in Lactobacillus colonization and an overgrowth of facultative anaerobic bacteria. Whilst vaginal inflammation is usually absent in BV, it is the most serious risk factor for women of reproductive age because of its association with adverse pregnancy outcomes such as preterm birth [11, 12], acquisition of sexually transmitted infections [13], and the development of pelvic inflammatory disease (PID) [14, 15]. Candida infection is the second most common RTI and VVC affects up to 75% of reproductive-age women at least once [16]. Candida infection can be asymptomatic and is thus often referred to as colonization. VVC, the disease state of Candida infection, is diagnosed by observing Candida species in presence of local signs and symptoms of inflammation [17]. TV infection is the most prevalent STI worldwide [18]. TV infection can lead to reproductive complications such as pelvic inflammatory disease, tubal factor infertility, pregnancy loss, premature membrane rupture, preterm delivery, low birth weight [18].

The clinical signs and symptoms of these infections overlap to a large extent, though the etiologies are quite different. Common clinical symptoms include vaginal discharge, dyspareunia, itching and burning sensation. However, the poor sensitivity and specificity of individual clinical symptoms, and the poor performance of standard laboratory tests in many settings, make the accurate clinical diagnosis of these infections difficult [19]. In addition, a significant proportion of genital infections are asymptomatic such that laboratory testing is required to diagnose and identify the specific cause.

Most studies assessing an association between MHM and RTI have used self-reported symptoms to measure outcomes which are likely biased [4]. When assessing MHM practices, most published studies have focused on absorbents, either comparing the type of absorbent used (e.g. reusable vs. disposable pads) or, in fewer cases, washing, drying and storing methods for cloths used for absorption or other personal hygiene practices [4, 20, 21].

In a recent case-control study in Odisha, India, it was observed that women of menstruating age with urinary tract infections (UTI) and BV were more likely using reusable absorbent pads than using disposable pads [22]. Both UTI and BV were also associated with a lack of a private space for changing, cleaning and washing during menstruation [22]. In that study, BV and UTIs were not detected in a significant proportion of women with vaginal symptoms suggesting that other urogenital infections may be responsible for these symptoms. Only one study has explored the association of VVC and TV infection with MHM practices, reporting that the use of saris for menstrual absorbent protection was safer than rags washed in river and dried at home [6]. However, no other MHM practices were explored in that study. In this study we address this gap by assessing the association of different MHM practices among women of reproductive age in Odisha with the risk of three common infections of the lower reproductive tract; Bacterial vaginosis (BV), Candida, and *Trichomonas vaginalis* (TV).

## Methods

### Study design and study population

This was a cross-sectional study that surveyed women attending the Obstetrics and Gynecology (O&G) Out-Patient Department (OPD) of two public hospitals in Odisha state, India: Capital Hospital, Bhubaneswar; and, Ispat General Hospital (IGH), Rourkela. Capital Hospital is a government hospital with approximately 700 beds specializing in Obstetrics and Gynaecology, and Family Welfare which caters to the urban and peri-urban population of Bhubaneswar city as well as adjoining rural areas. Almost all health care services are provided free of cost and the majority of patients attending Capital hospital are from the financially disadvantaged groups specifically from the urban and peri urban slums and rural areas IGH, Rourkela is managed by the Steel Authority of India Limited and provides free treatment for its employees and their dependents and treatment for the general public at a subsidized cost. The hospital is situated in a District inhabited predominantly by a tribal population, a large proportion of the patients attending the hospital are from tribal populations. The study population in Rourkela is therefore, covers a broad spectrum of socio-demographic status. The patient recruitment was done from April 2015 to February 2016.

Non-pregnant women of reproductive age (18–45 years) attending the O&G OPD with vaginal symptoms (vaginal discharge, itching, burning, dyspareunia, with lower abdominal pain, and lower back pain), or attending the Family Welfare Department (FWD) for contraceptives, such as intra-uterine devices (IUD) were eligible for enrolment. We excluded all women meeting one or more of the following criteria: menstruating during the clinic visit, had a hysterectomy, taken a course of antibiotics during the previous three weeks, used oral contraceptive pills in the previous three months, had diabetes mellitus, were HIV positive, or had any severe medical disorders requiring immediate referral to a higher level of health care.

### Data collection

**Fig. 1:**
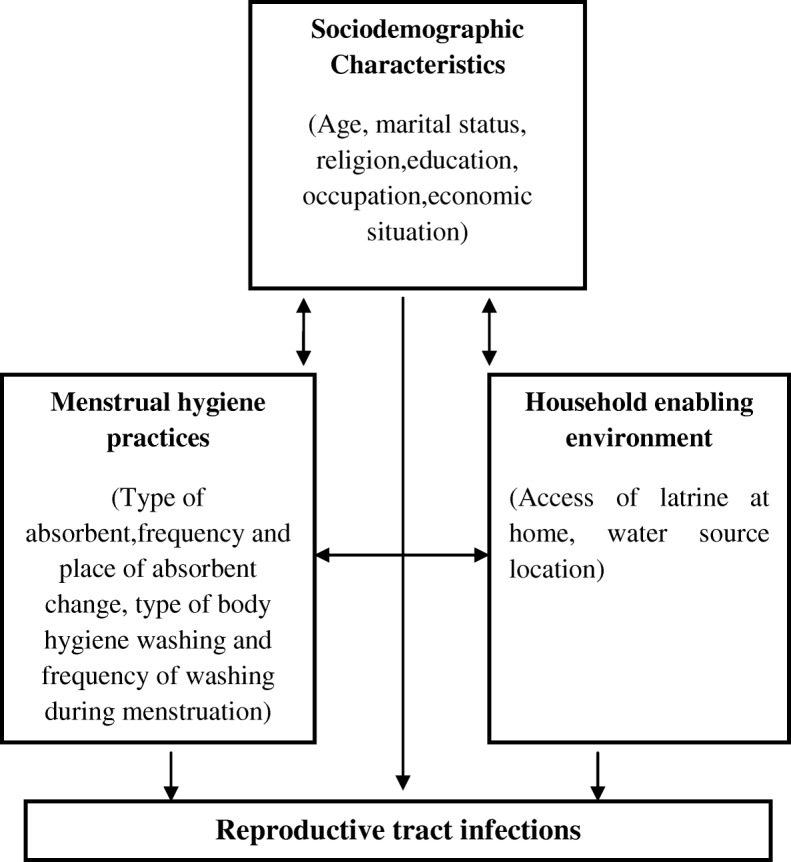
Conceptual framework of risk factors. A conceptual framework with three groups of risk factors: sociodemographic characteristics, MHM practices and WASH access variables

After identification by the treating doctor, all potential study participants underwent an informed consent process in the local language (Odia) and those choosing to participate provided written consent. The participants were informed that they would be tested for RTI and that their results would be provided free of cost and confidentially. Trained female interviewers administered a questionnaire collecting information about potential risk factors for the three infections. The examined risk factors were selected using a pre-specified conceptual framework with three groups of risk factors: socio-economic characteristics, MHM practices (related with menstrual absorbent material and body hygiene), and WASH access variables (access to water and sanitation resources for practicing safe MHM) (Fig. 1). This framework was already used by the authors in other related work, as it captures aspects of the working definition of MHM by the Joint Monitoring Program of the WHO and UNICEF in 2012 (defining MHM as: *Women and adolescent girls using a clean menstrual management material to absorb blood that can be changed in privacy as often as necessary for the duration of the menstruation period, using soap and water for washing the body as required, and having access to facilities to dispose the used menstrual management materials*) and also other important risk factors related to the RTI identified in the literature. We used a questionnaire developed and tested previously by our team [22]. Information on socioeconomic factors included age, marital status, religion, education, occupation, number of family member and monthly income. Information on MHM practices included the type of menstrual absorbent used, individual hygiene habits, and domestic water and sanitation conditions. Questions on the use of absorbents included: the type of absorbent (reusable/disposable), the type of material used in reusable absorbents, frequency and place of change absorbent. In the case of reusable absorbents, information on the method of washing, drying, packaging, and storing were also collected. Information on water and sanitation conditions in their households, include information about access to latrine and place where the main water source is located. Clinical symptoms related to vaginal discharge (its quantity, smell, colour, and consistency), burning or itching of the genitalia, and the presence of ulcers at vulva and labia, vesicles, papules were collected (Additional file 1).

### Collection of vaginal samples

A speculum examination of the vagina and cervix was done by a trained O&G specialist to assess the presence of cervical erythema, bleeding, inflammation, with or without discharge, and ulcers at cervix. Vaginal specimens from the posterior vaginal fornix were collected using four BD BBL swabs (BD, Maryland, USA). The first swab was used for Gram staining to diagnose BV, the second swab was used to determine the presence of Candida, the third swab was used for DNA extraction for diagnosis of TV by nucleic acid amplification test, and the fourth swab was stored for future studies. After labelling, all materials (slides and swabs) were transported immediately to a local laboratory for preservation and subsequent diagnosis.

### Diagnosis of BV, TV, and Candida infection

BV was diagnosed using Nugent’s laboratory diagnostic criteria [23] and a Nugent score (NS) between 0 and 10 was generated using the Nugent criteria. A NS of 0–3 was interpreted as normal or negative for BV, a score of 4–10 as intermediate/abnormal for BV or positive for BV. Presence of TV was identified by nucleic acid amplification tests [24] and diagnostic PCR was used to ascertain TV infection. Lastly, AlbiQuick™ rapid test (HARDY Diagnostic, CA, USA) was used for identification of *Candida albicans* as per manufacturer’s instructions. (More details for diagnosis of the three infections in Additional file 2).

### Data handling and statistical analysis

All data were double-entered into Epi Info 7 software (*Epi info, Centers for Disease Control and Prevention (CDC), Atlanta, USA*) and analyzed using Stata 11.0 (StataCorp, *Stata Statistical Software: Release 11*. 2011, StataCorp LP: College Station, Texas, USA). Pearson χ2 tests were used for initial examination of the association between individual exposures and each of the four of outcomes of interest (BV, Candida Infection, TV and having at least one infection). Among the participant-subgroup reporting use of reusable pads, we explored whether different washing, drying and storing practices were associated with the three studied infections. To estimate the prevalence rate ratios (PRR) and adjusted prevalence rate ratios (aPRR), and the associated 95% confidence intervals (CIs) for factors in relation to each of the four outcomes we used Poisson survival regression models.

Potential risk factors for the three infections were examined using a pre-specified conceptual framework with three groups of risk factors: socio-demographic characteristics, MHM practices (related with menstrual absorbent material and body hygiene), and WASH access variables (access to water and sanitation resources for practicing safe MHM) (Fig. 1). Analysis was done using a hierarchical approach. Age and education were retained in all final models as a priori potential confounding factors. Other variables were selected for inclusion based on stepwise elimination of variables associated (*P* < 0.1) in univariate analysis with the outcomes of interest. Owing to the collinearity of MHM practices, the effect of each practice on the risk of infection was evaluated in separate multivariable models adjusted for age, and education and by the other variables of interest from univariate model. Potential interacting variables were evaluated by including an interaction term in the regression model which was retained if judged statistically significant (*P* < 0.05) by likelihood-ratio test.

## Results

**Fig. 2:**
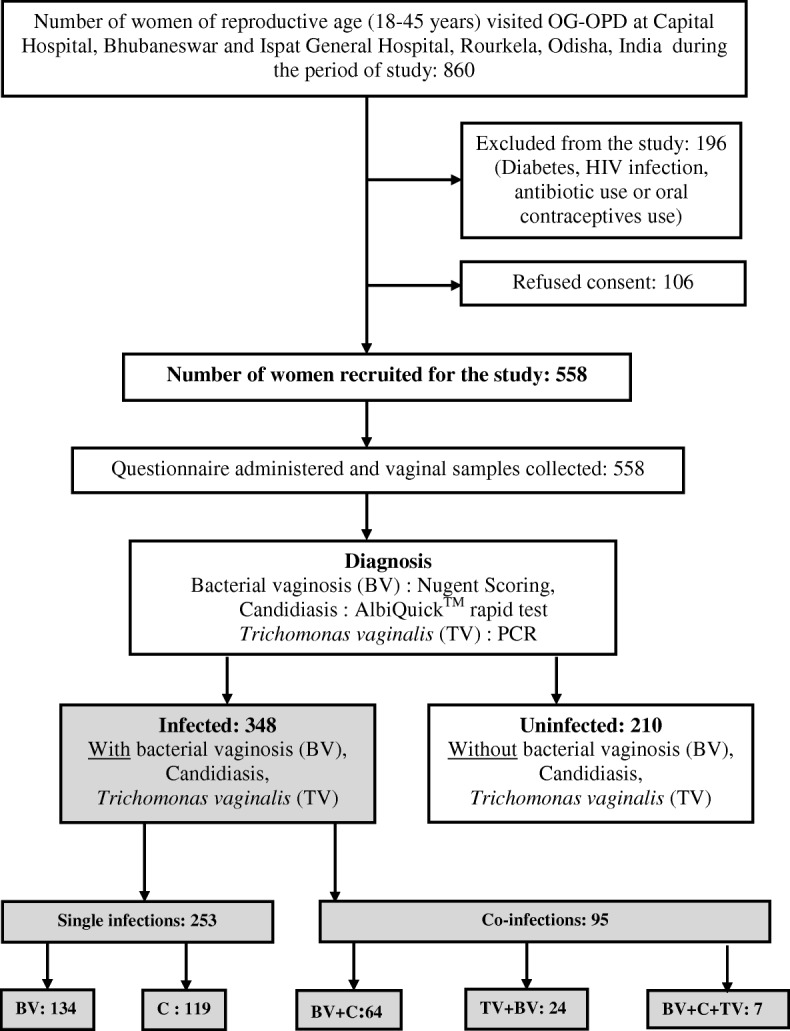
Schematic diagram of recruitment of participants. Schematic diagram of recruitment of participants at Capital Hospital, Bhubaneswar and Ispat General Hospital, Rourkela, Odisha, India

Of 860 reproductive age women who visited the gynaecology OPD of the two hospitals during the study period 558 were enrolled in the study, 106 did not consent to participate and a further 196 were excluded according to our criteria (Fig. 2). Among enrolled women50.5% of women had at least one RTI symptom (data not shown). 29% of infected women reported that the symptoms motivating attendance at the clinic were recurrent, but only 30 out of 101 changed their MHM practices and only 17 changed the type of absorbents (the majority of them using disposable ones when interviewed) as a result of symptoms (Data not shown). Having abnormal vaginal discharge was the most frequently reported symptom (36.6%), followed by the feeling of burning or itching in the genitalia (27.9%) and presence of genital sores (8.9%) (data not shown). 62.4% were diagnosed with at least one of the three tested infections (1.3% had all three infections, 15.8% had two infections and 45.3% had only one infection). BV was the most prevalent infection (41%), followed by Candida (34%) and TV (5.6%) (Table 1).

**Table 1:** Socioeconomic characteristic of women participating in the study according to infection status Data are expressed in no (%) of women

Exposures	With *BV* (*N* = 229)	Without *BV* (*N* = 329)	*P*-value	With *Candidiasis* (*N* = 190)	*Without Candidiasis (N = 368)*	*P*-value	With TV (*N* = 31)	Without TV (*N* = 527)	*P*-value
Age									
18–25 years	63 (27.51)	100 (30.40)	0.758	59 (31.05)	104 (28.26)	0.069	7 (22.58)	156 (29.60)	0.468
26–35 years	89 (38.86)	124 (37.69)		81 (42.63)	132 (35.87)		15 (48.39)	198 (37.57)	
36–45 years	77 (33.62)	105 (31.91)		50 (26.32)	132 (35.87)		9 (29.03)	173 (32.83)	
Marital status									
Never married	23 (10.04)	52 (15.81)	0.146	28 (14.74)	47 (12.77)	0.137	1(3.23)	74 (14.04)	0.177
Married	203(88.65)	273 (82.98)		162 (85.26)	314 (85.33)		30 (96.77)	446 (84.63)	
Divorcee& widow	3 (1.31)	4 (1.22)		0	7 (1.90)		0	7 (1.33)	
What is your religion									
Hindu	222(96.94)	316 (96.05)	0.098	183 (96.32)	355 (96.47)	0.524	31 (100.00)	507 (96.20)	0.543
Muslim	7 (3.06)	7 (2.13)		6 (3.16)	8 (2.17)		0	14 (2.66)	
Christian	0	6 (1.82)		1 (0.53)	5 (1.36)		0	6 (1.14)	
What is your caste or tribe									
Scheduled caste (SC)	41 (18.47)	47 (14.87)	0.250	22 (12.02)	66 (18.59)	0.067	5 (16.13)	83 (16.37)	0.667
Scheduled tribe (ST)	6 (2.70)	10 (3.16)		4 (2.19)	12 (3.38)		0	16 (3.16)	
Other backward caste (OBC)	127 (57.21)	168 (53.16)		114 (62.30)	181 (50.99)		16 (51.61)	279 (55.03)	
Other Caste	48 (21.62)	91 (28.80)		43 (23.50)	96 (27.04)		10 (32.26)	129 (25.44)	
Education									
No formal education	40 (17.47)	46 (13.98)	0.266	40 (21.05)	46 (12.50)	**0.006**	7 (22.58)	79 (14.99)	0.235
5th – 10th grade	102 (44.54)	137 (41.64)		85 (44.74)	154 (41.85)		9 (29.03)	230 (43.64)	
12th grade or higher	87 (37.99)	146 (44.38)		65 (34.21)	168 (45.65)		15 (48.39)	218 (41.37)	
What is your occupation									
Employed	30 (13.10)	52 (15.81)	0.351	26 (13.68)	56 (15.22)	0.642	4 (12.90)	78 (14.80)	0.534
Housewife	179 (78.17)	238 (72.34)		141 (74.21)	276 (75.00)		26 (83.87)	391 (74.19)	
Student	15 (6.55)	33 (10.03)		20 (10.53)	28 (7.61)		1 (3.23)	47 (8.92)	
Other	5 (2.18)	6 (1.82)		3 (1.58)	8 (2.17)		0	11 (2.09)	
How many family members in hh									
3 or less	47 (20.52)	66 (20.06)	0.712	44 (23.16)	69 (18.75)	0.470	7 (22.58)	106 (20.11)	0.379
4–5	119 (51.97)	162 (49.24)		92 (48.42)	189 (51.36)		12 (38.71)	269 (51.04)	
More than 5	63 (27.51)	101 (30.70)		54 (28.42)	110 (29.89)		12 (38.71)	152 (28.84)	
Economic status (monthly income)									
< 5000.00	30 (13.10)	35 (10.64)	0.427	26 (13.68)	39 (10.60)	0.607	5 (16.13)	60 (11.39)	0.776
5000.00 – 10,000	65 (28.38)	114 (34.64)		59 (31.05)	120 (32.61)		11 (35.48)	168 (31.88)	
> 10,000.00	103 (44.98)	136 (41.34)		77 (40.53)	162 (44.02)		11 (35.48)	228 (43.26)	
Don’t know	31 (13.54)	44 (13.37)		28 (14.74)	47 (12.77)		4 (12.90)	71 (13.47)	

*P*-value determined by Pearson’s chi-square (χ^2^) test. (hh = household)

The median age of participants was 31 (range 18–45), and 85.3% of women were married and most identified as Hindu (96.4%). 15.4% of women had no formal education, 42% had attained 12th grade (12 years of education) or higher education. Table 1 shows the socioeconomic characteristics of study participants by the three infections. A significant difference in the education status (*P* < 0.006) between those with Candida and those without was observed, while other demographic and socio-economic variables didn’t show any significant difference among this group or any of the other infections.

Univariate analysis showed that certain MHM practices were associated with certain infections (Table 2). Women with BV or Candida infections were more likely to use reusable cloths more frequently than those without these infections (*P* < 0.014 and < 0.001, respectively). However, there was no observed difference by type of reusable material (Table 2). Similarly, women with BV or Candida infections were more likely to use reusable cloths versus disposable pads, to change their absorbents somewhere other than in a toilet facility, and to wash less frequently during menstruation. Women changing absorbents less frequently were more likely to have a BV infection but not a Candida infection. TV infection was not associated with any of the tested variables, and access to sanitation facilities or water at the household level was not associated with any of the three infections.

**Table 2:** Different menstrual hygiene management practices and WASH access variables of women participating in the study according to infection status

Exposures	*With BV (N = 229)*	*Without BV (N = 329)*	*P-value*	*With Candidiasis(N = 190)*	*Without Candidiasis (N = 368)*	*P-value*	*With TV (N = 31)*	*Without TV (N = 527)*	*P*-value
MHM practices									
Absorbent material									
Disposable sanitary pads	99 (43.23)	177 (53.80)	**0.014**	70 (36.84)	206 (55.98)	**0.001**	12 (38.71)	264 (50.09)	0.218
Reusable cloths	130 (56.77)	152 (46.20)		120 (63.16)	162 (44.02)		19 (61.29)	263 (49.91)	
Type of reusable material use									
Old cotton fabric	61 (46.92)	84 (55.26)	0.162	62 (51.67)	83 (51.23)	0.943	9 (47.37)	136 (51.71)	0.715
Old silk/nylon fabric	69 (53.08)	68 (44.74)		58 (48.33)	79 (48.77)		10 (52.63)	127 (48.29)	
Frequency of change of absorbent									
Once a day	69 (30.13)	60 (18.24)		44 (23.16)	85 (23.10)	0.869	7 (22.58)	122 (23.15)	0.653
Twice a day	110 (48.03)	156 (47.42)	**0.001**	88 (46.32)	178 (48.37)		17 (54.84)	249 (47.25)	
Three times a day or more	50 (21.83)	113 (34.35)		58 (30.53)	105 (28.53)		7 (22.58)	156 (29.60)	
Place of change of absorbent									
In a toilet facility	130 (56.77)	218 (66.26)	**0.023**	112 (58.95)	236 (64.13)	0.231	20 (64.52)	328 (62.24)	0.799
Outside a toilet facility	99 (43.23)	111 (33.74)		78 (41.05)	132 (35.87)		11 (35.48)	199 (37.76)	
What type of washing do you practice during menstruation									
Only vaginal wash	45 (19.65)	58 (17.63)	0.545	43 (22.63)	60 (16.30)	0.068	5 (16.13)	98 (18.60)	0.731
Bath of full body	184 (80.35)	271 (82.37)		147 (77.37)	308 (83.70)		26 (83.87)	429 (81.40)	
Frequency of washing during menstruation									
Twice or more per day	96 (41.92)	166 (50.46)	**0.047**	77 (40.53)	185 (50.27)	**0.029**	15 (48.39)	247 (46.87)	0.869
Once a day	133 (58.08)	163 (49.54)		113 (59.47)	183 (49.73)		16 (51.61)	280 (53.13)	
WASH Access variables									
Do you have a latrine at home?									
Yes	186 (81.2)	265(80.6)	0.84	162 (85.3)	289 (78.5)	0.056	25 (80.7)	426 (80.8%)	0.97
No	43 (18.8)	64 (19.4)		28 (14.7)	79 (21.5)		6 (19.3)	101 (19.2%)	
Where is the water source located?									
In the house	155 (67.7)	211 (64.1)	0.14	122 (64.2)	244 (66.3)	0.74	20 (64.5)	346 (65.7)	0.64
In the yard	34 (14.9)	42 (12.8)		24 (12.6)	52 (14.1)		6 (19.4)	70 (13.3)	
At relatives/neighbor house or yard	2 (3.65)	2 (0.87)		6 (3.2)	8 (2.2)		0 (0)	14 (2.7)	
At a public location	38 (16.6)	64 (19.4)		38 (20)	64 (17.4)		5 (16.1)	97 (18.4)	

Data are expressed in no (%) of women. *P*-value determined by Pearson’s chi-square (χ^2^) test

We used multivariable analysis to assess the association between MHM variables and the three outcomes of interest whilst controlling for potential confounding factors. Reusable cloths were found to strongly associated with Candida infection (aPRR = 1.54, 95%CI 1.2–2.0), weakly associated with BV infection (aPRR =1.23, 95%CI 1.0–1.54) and not associated with TV infection (Table 3). Changing the absorbent in a location other than in a toilet facility was associated with a higher risk of BV (aPRR =1.21, 95%CI 1.0–1.48). Women who washed themselves less often during menstruation had a higher risk of BV and Candida infection (aPRR =1.25, 95%CI 1.0–1.55, aPRR =1.34, 95%CI 1.07–1.7, respectively) but not for TV. In the BV confirmed group, women who changed their absorbent material more frequently (twice or more) presented with reduced rates of infections compared with women who changed only once per day (aPRR = 0.75, 95%CI 0.6–0.93, aPRR =0.56, 95%CI 0.4–0.75, respectively).

**Table 3:** Multivariable table with crude and adjusted PRR for each disease model

Risk Factors	*BV Unadjusted*	*BV Adjusted*	*Candidiasis Unadjusted*	*Candididasis Adjusted*	*TV Unadjusted*	*TV Adjusted*	Infected Unadjusted	Infected Adjusted
	PRR *(95%CI)*	PRR*(95%CI)*	PRR *(95%CI)*	PRR *(95%CI)*	PRR*(95%CI)*	PRR *(95%CI)*	PRR(95%CI)	PRR(95%CI)
Absorbent material								
Disposable	1.0	1.0	1.0	1.0	1.0	1.0	1.0	1.0
Reusable	1.28 (1.1–1.6)	1.23(1.0–1.54)	1.67(1.3–2.1)	1.54(1.21–2.0)	1.55(0.8–3.1)	1.78(0.81–3.9)	1.38(1.2–1.6)	1.35 (1.17–1.56)
Frequency of change of absorbent								
Once a day	1.0	1.0	1.0	1.0	1.0	1.0	1.0	1.0
Twice a day	0.77 (0.6–0.9)	0.75(0.6–0.93)	0.96(0.7–1.3)	0.97(0.7–1.27)	1.17(0.5–2.8)	1.12(0.47–2.7)	0.81 (0.7–0.9)	0.79 (0.60–0.9)
More than twice a day	0.57 (0.4–0.8)	0.56(0.4–0.75)	1.04(0.8–1.4)	1.08(0.78–1.5)	0.79(0.3–2.2)	0.74(0.26–2.1)	0.74 (0.6–0.9)	0.72 (0.61–0.86)
Place of change absorbent								
In a toilet facility	1.0	1.0	1.0	1.0	1.0	1.0	1.0	1.0
Outside a toilet facility	1.26 (1.03–1.53)	1.21(1.0–1.48)	1.15 (0.91–1.45)	1.09(0.8–1.3)	0.91(0.4–1.8)	0.81(0.4–1.64)	1.24(1.19–1.4)	1.16 (1.02–1.31)
Frequency of washing during menstruation								
Twice or more per day	1.0	1.0	1.0	1.0	1.0	1.0	1.0	1.0
Once a day	1.22 (1.0–1.5)	1.25(1.0–1.55)	1.29(1.0–1.6)	1.34(1.07–1.7)	0.94(0.5–1.9)	1.09(0.53–2.3)	1.18(1.0–1.4)	1.21(1.06–1.38)

*PRR* Prevalence rate ratio, *CI* confidence interval

In order to explore if these MHM practices and other exposure factors were also associated with at least one infection, we performed univariate and multivariate analysis between the same exposure variables and the new infected outcome group (Table 3). Analysis of the aggregated infection outcome (one or more of any infection) showed a similar pattern in the adjusted multivariate analysis with significant association between at least one infection and reusable absorbents (aPRR = 1.35, 95%CI 1.17–1.56), non-access to a toilet facility for changing the pads (aPRR = 1.16, 95%CI 1.02–1.31) and lower frequency of self-washing and cleaning during menstrual cycle (aPRR = 1.21, 95%CI 1.06–1.38). Higher frequency of change of absorbents was protective of infections (aPRR = 0.72, 95%CI 0.61–0.86).

Use of reusable pads was correlated with other practices that plausibly influence the risk of RTI which required further investigation to assess what aspects of reusable pad use might explain the higher observed risk among users. In a sub-group of women who reported using reusable cloths (*n* = 282), we analyzed the independent association of the method of washing, the place of washing, the place of drying, the method of packing for storage, and the place of storage of the reusable pads on different infections (Table 4). Almost all women washed the reusable menstrual cloths in soap and water (data not shown) and there was no difference in risk of at least one infection by place of washing nor method of packing for storage of pads. However, significant differences in Candida infection were associated with the place of drying and the place of storage of the cloths. Women who dried their reusable menstrual absorbent inside their house and women who kept the stored cloth hidden in the toilet compartment were more likely to have Candida infections compared with women who dried them in the sun or open space (aPRR = 1.78, 95%CI 1.34–2.38) or who kept stored within cupboard in the changing room (aPRR = 1.96, 95%CI 1.49–2.57).

**Table 4:** Multivariable table among women who use reusable cloth comparing the PRR of different MHM practices

Risk Factors	*BV Unadjusted*	*BV Adjusted*	*Candidiasis Unadjusted*	*Candidiasis Adjusted*	*TV Unadjusted*	*TV Adjusted*
	PRR *(95% CI)*	PRR *(95% CI)*	PRR *(95% CI)*	PRR *(95% CI)*	PRR *(95% CI)*	PRR *(95% CI)*
Place to wash absorbent						
Inside the toilet stall	1.0	1.0	1.0	1.0	1.0	1.0
At the tube well or yard	1.04(0.81–1.34)	1.01 (0.76–1.34)	0.80 (0.61–1.07)	0.86 (0.64–1.16)	1.39 (0.58–3.33)	2.30 (0.80–6.56)
After washing, how did you dry the cloth						
Dry it in the Sun/open space	1.0	1.0	1.0	1.0	1.0	1.0
Dry it inside the house	1.15(0.89–1.48)	1.07(0.82–1.39)	1.89(1.41–2.55)	1.78(1.34–2.38)	2.13(0.83–5.47)	1.91(0.68–5.32)
How do you store the cloth for use next time						
Wrapped in polythene	1.0	1.0	1.0	1.0	1.0	1.0
Wrapped in another material	1.01(0.76–1.35)	1.04(0.76–1.42)	0.83(0.59–1.16)	0.86(0.62–1.20)	0.33(0.79–1.42)	0.28(0.07–1.10)
Where did you store the cloth for use next time						
Within the cupboard in the changing room	1.0	1.0	1.0	1.0	1.0	1.0
In the toilet	1.10(0.85–1.42)	1.11(0.85–1.43)	1.98(1.50–2.61)	1.96(1.49–2.57)	0.63(0.24–1.61)	0.54(0.21–1.38)

*PRR* Prevalence rate ratio, *CI* confidence interval

## Discussion

Among the women studied, the RTI prevalence was 62.4% with BV the most common infection (41%) and abnormal vaginal discharge the most commonly reported RTI symptom. These results are slightly higher than those observed in the District Level Household Survey - Reproductive & Child Health (DLHS-RCH) (2002–4) survey which reported a 35.2% prevalence of RTI/STIs in Odisha or the ones reported in another district of Odisha (39.2%) [25]. This difference may be due to the use of syndromic diagnosis of RTI/STI cases or because our study was done in an outpatient hospital setting where attending women have health problems. However, the frequencies of these infections are similar to those reported in other Indian studies [26, 27]. Infectious causes of vaginal discharge were observed in 51.75% women with BV 26.25%, Candidiasis 15.25%, Trichomoniasis 12.3%, and mixed infections 5.5% [28].

The current study supports the hypothesis that certain MHM practices are associated with a higher risk of lower reproductive tract infections. Women using reusable absorbent pads were more likely to have Candida infection and BV than women using disposable pads. This effect remains statistically significant for Candida infection, but not for BV, after adjustment for potential confounding factors. A similar lack of association with BV was observed in a previous study by our team [22] and also in an intervention study conducted in Kenya which showed that distribution of disposable sanitary pads among school girls did not reduce the risk of BV [29, 30].

The place where women normally change their absorbent material was also associated with BV after adjusting for confounders (changing inside a toilet facility was found to be protective for these infections). Similar findings with BV were observed in our previously hospital based study [22]. The current study cannot ascertain whether this association is causal, but it suggests that having a secure, comfortable, and hygienic place where women can practice MHM without stress and with access to water is important. This finding is supported by another community level related study that we conducted in Odisha, which showed that RTI self-reported symptoms were less common in women using a latrine for defecation versus open defecation [31].

Interestingly, higher education was found to be protective only for Candida infection after adjustment for confounding (data not shown). Many different studies have showed that with education, people are better prepared to prevent disease and use health services effectively [22, 32, 33] but the reasons why this factor only affects Candida infection and not BV or TV are unknown.

More frequent changing of absorbents and regular body washing during menstruation was associated with a lower risk of BV which was not observed in our previous study in the same setting [22]. The difference could be due to the different diagnostic criteria used for BV in this study, whilst the earlier study used Amsel’s clinical criteria, this study used a more sensitive laboratory-based Nugent’s criteria for diagnosis of BV. We are not sure about the mechanism how these exposures are responsible for BV, but it can be presumed that accumulation of blood in the vagina for a prolonged period of time may influence the vaginal ecosystem. The similar findings in the Kenyan study that girls given disposable sanitary pads tend to share them with others, and they use their remaining pads for a longer duration of time, adds plausibility to this hypothesis [30]. BV is caused by an imbalance of vaginal microbiota and the composition of vaginal microflora can be influenced by several factors, including ethnicity [34], sexual activity [35], and host immune response [36], especially the local vaginal mucosal response. In addition, it has been observed that the rectal microflora can serve as a reservoir for colonization of the vaginal ecosystem [37]. Therefore, it is logical to assume that MHM practices may influence the risk of vaginal infections. Another recent cross-sectional study in India, showed an association between lower frequency of genitalia washing associated with higher self-reported symptoms of itching and pustules in the genitalia [38].

Unhygienic MHM practices may create abnormally moist conditions in the vulvo-vaginal area, which may promote opportunistic infections such as Candida. Once infected, removal of Candida from the clothes may be difficult in absence of adequate cleaning and drying. In this study, whilst both BV and Candida infection were associated with several unhygienic MHM practices, TV infection was not. This could be due to the generally low prevalence of this infection among this population [39] but may also be due to the fact that TV is primarily transmitted through sexual contact. The intervention study mentioned above, showed that the distribution of menstrual cups or sanitary pads was associated with a lower prevalence of STIs (*C. trachomatis* and TV, in particular). The authors hypothesized this may have prevented STIs as girls in the study population had previously reported having sex to obtain money to buy MHM materials [30].

There are several limitations to our study. Firstly, this was a hospital-based study and socio-economic, education, and WASH characteristics risk factors may differ from the wider population. Only women attending the Obstetrics and Gynecology departments consented to participate in the study, and to provide vaginal swabs, such that our findings may not be representative of either the wider population attending other sections of the hospital and, again, limiting the generalizability of our findings to the wider population. Secondly, this is a cross-sectional observational study and so we cannot infer causality based on the observed associations between MHM practices and health outcomes. Although we adjusted for a range of potential confounders, it is likely that there is residual confounding in our results. Furthermore, we did not collect data on, nor therefore adjust for, other possibly important factors, such as sexual practices or other infections. The Candida culture was able to detect only *Candida albicans* but no non-albican species. There is a possibility that other forms of Candida were not detected.

Despite these limitations, this is the first study, to our knowledge, to collect detailed information on individual MHM practices linked to individual laboratory confirmed lower RTI. The strengths of our work include: a large sample size with power to test associations between MHM practices and individual RTIs; the use of validated laboratory methods with external quality controls to diagnose RTI; and, lastly, the use of exclusively female doctors and interviewers in the study to facilitate participation in the study.

In India, and globally, there has been significant interest in recent years around the issue of safe MHM, and much discussion around the inclusion of MHM in the post-2015 sustainable development goals [40]. One intervention that has been advocated is the provision of disposable pads [41] but many limitations have been identified. Commercially available, disposable pads are often too expensive for poor communities [42, 43] and the promotion of disposable pads alone is unlikely to fully address the health and other problems associated with poor MHM. Beyond the prohibitive cost, misconceptions exist that disposable pads possess high absorbing capacity leading to reduced frequency of change, and accumulation of blood over a longer period of time. Finally, safe disposal of commercial pads is a challenge, which has not been addressed in many countries [44].

Reusable materials are still used widely and, for them to be hygienic, safe accompanying hygienic practices are required. This can be a great challenge in some settings such as high density urban settlements where private space for such practices is limited and in low income rural areas where the water supply is limited.

Whilst this study aimed to assess the risk presented by certain MHM practices for the health of women, it is important to recognize that access to safe, affordable and appropriate MHM is as much an issue of social justice as it one of public health [33, 40]. Whilst our study suggests that certain MHM practices contribute to the RTI disease burden, the justification for supporting all girls and women to manage their menstruation with safety, dignity, and comfort must extend beyond this public health rationale. Our study does though provide important insights as to which aspects of MHM might be considered for prioritization in national and global policy concerning MHM.

## Conclusion

In conclusion, the results of our study demonstrate a strong association between poor MHM practices and two lower RTIs: BV and *Candida albicans* infection. Our findings support global calls for a dual focus on the provision of appropriate absorbent materials as well as ensuring that environmental conditions that enable associated hygiene including washing and drying of pads can be practiced with safety and dignity by women. Future research efforts in this area should seek to measure whether and, to what extent, such interventions reduce the risk of RTI through well-conceived and well-designed intervention studies.

## Additional files

Additional file 1: Variable table with Definitions (Table that present all the variables measured in the study and their definitions). (DOC 62 kb)

Additional file 2: Diagnosis of BV, TV, and Candida infection. Detail explanation about laboratory methods used to diagnosed the studied infections [45, 46]. (DOCX 12 kb)

Additional file 3: Strobe checklist for Cross-sectional studies (Strobe checklist requested from reviewers. It is not referred in the text, but it is added as Additional file 3). (DOC 97 kb)

## Abbreviations

aPRR: Adjusted prevalence rate ratios
BV: Bacterial vaginosis
CI: Confidence interval
FWD: Family Welfare Department
IUD: Intra-uterine devices
MHM: Menstrual hygiene management
OPD: Out-patient department
PID: Pelvic inflammatory disease
PRR: Prevalence rate ratios
RTI: Reproductive tract infection
STI: Sexually transmitted infection
TV: *Trichomonas vaginalis*
UTI: Urinary tract infections
VVC: Vulvo-vaginal candidiasis

## References

[1] Baisley K, Changalucha J, Weiss HA, Mugeye K, Everett D, Hambleton I (2009). Bacterial vaginosis in female facility workers in North-Western Tanzania: prevalence and risk factors. Sex Transm Infect.

[2] Aniebue UU, Aniebue PN, Nwankwo TO (2009). The impact of pre-menarcheal training on menstrual practices and hygiene of Nigerian school girls. Pan Afr Med J.

[3] Marni S, Sahin M, Paloubis L, Truong M, Sinden J (2016). WASH in schools empowers girls’ education: proceedings of the menstrual hygiene Management in Schools Virtual Conference 2015, United Nations Children’s fund and Columbia University. New York.

[4] Sumpter C, Torondel B (2013). A systematic review of the health and social effects of menstrual hygiene management. PLoS One.

[5] Hulland KR, Chase RP, Caruso BA, Swain R, Biswal B, Sahoo KC, Panigrahi P, Dreibelbis R (2015). Sanitation, stress, and life stage: a systematic data collection study among women in Odisha, India. PLoS One.

[6] Wasserheit JN, Harris JR, Chakraborty J, Kay BA, Mason KJ (1989). Reproductive tract infections in a family planning population in rural Bangladesh. Stud Fam Plan.

[7] Bhatti LI, Fikree FF (2002). Health-seeking behavior of Karachi women with reproductive tract infections. Soc Sci Med.

[8] Desai GS, Patel R (2011). Incidence of reproductive tract infections and sexually transmitted diseases in India: levels and differentials. J Fam Welf.

[9] Mishra S, Dasgupta D, Ray S (2016). 2016. A study on the relationship of sociocultural characteristics, menstrual hygiene practices and gynaecological problems among adolescent girls in eastern India. Int J Adolesc Med Health.

[10] Mitchell H (2004). Vaginal discharge - causes, diagnosis, and treatment. BMJ.

[11] Nelson DB, Bellamy S, Nachamkin I, Ness RB, Macones GA, Allen-Taylor L (2007). First trimester bacterial vaginosis, individual microorganism levels, and risk of second trimester pregnancy loss among urban women. Fertil Steril.

[12] Hillier SL, Nugent RP, Eschenbach DA, Krohn MA, Gibbs RS, Martin DH (1995). Association between bacterial vaginosis and preterm delivery of a low-birth-weight infant. N Engl J Med.

[13] Wiesenfeld HC, Hillier SL, Krohn MA, Landers DV, Sweet RL (2003). Bacterial vaginosis is a strong predictor of Neisseria gonorrhoeae and chlamydia trachomatis infection. Clin Infect Dis.

[14] Ness RB, Kip KE, Hillier SL, Soper DE, Stamm CA, Sweet RL, Rice P, Richter HE (2005). A cluster analysis of bacterial vaginosis—associated microflora and pelvic inflammatory disease. Am J Epidemiol.

[15] Hillier SL, Kiviat NB, Hawes SE, Hasselquist MB, Hanssen PW, Eschenbach DA, Holmes KK (1996). Role of bacterial vaginosis associated microorganisms in endometritis. Am J Obstet Gynecol.

[16] Sobel JD, Faro S, Force RW, Foxman B, Ledger WJ, Nyirjesy PR, Reed BD, Summers PR (1998). Vulvovaginal candidiasis: epidemiologic, diagnostic, and therapeutic considerations. Am J Obstet Gynecol.

[17] Sobel JD (2007). Vulvovaginal candidosis. Lancet.

[18] Fichorova RN (2009). Impact of T. vaginalis infection on innate immune responses and reproductive outcome. J Reprod Immunol.

[19] Anderson MR, Klink K, Cohrssen A (2004). Evaluation of vaginal complaints. JAMA.

[20] Bhatia JC, Cleland J (1995). Self-reported symptoms of gynecological morbidity and their treatment in South India. Stud Fam Plan.

[21] Narayan K, Srinivasa D, Pelto P, Veerammal S (2001). Puberty rituals reproductive knowledge and health of adolescent schoolgirls in South India. Asia Pac Popul J.

[22] Das P, Baker KK, Dutta A, Swain T, Sahoo S, Das BS, Panda B, Nayak A, Bara M, Bilung B, Mishra PR, Panigrahi P, Cairncross S, Torondel B (2015). Menstrual hygiene practices, WASH access and the risk of urogenital infection in women from Odisha, India. PLoS One.

[23] Nugent RP, Krohn MA, Hillier SL (1991). Reliability of diagnosing bacterial vaginosis is improved by a standardized method of gram stain interpretation. J Clin Microbiol.

[24] Conrad M, Zubacova Z, Dunn AL, Upcroft J, Sullivan AS, Tachezy J, Carlton JM (2011). Microsatellite polymorphism in the sexually transmitted human pathogen trichomonas vaginalis indicates a genetically diverse parasite. Mol Biochem Parasitol.

[25] Panda S, Bebarrta S, Parida S, Panigrahi O (2007). Prevalence of RTI/STI among women of reproductive age group in district Sundergarh. Orissa Indian Journal of Practicing Doctor.

[26] Nagarkar A, Mashkar P (2015). A systematic review on the prevalence and utilization of health care services for reproductive tract infections/sexually transmitted infections: evidence from India. Indian J Sex Transm Dis.

[27] Sood S, Mohanty S, Kapil A, Tolosa J, Mittal S (2007). In pouch TV culture for detection of *Trichomonas vaginalis*. Indian J Med Res.

[28] Sivaranjini R, Jaisankar TJ, Thappa DM, Kumari R, Chandrasekhar L, Malathi M, Parija SC, Habeebullah S (2013). Spectrum of vaginal discharge in a tertiary care setting. Trop Parasitol.

[29] Juma J, Nyothach E, Laserson KF, Oduor C, Arita L, Ouma C, Oruko K, Omoto J, Mason L, Alexander KT, Fields B, Onyango C, Phillips-Howard PA (2017). Examining the safety of menstrual cups among rural primary school girls in western Kenya: observational studies nested in a randomised controlled feasibility study. BMJ Open.

[30] Phillips-Howard PA, Nyothach E, Ter Kuile FO, Omoto J, Wang D, Zeh C, Onyango C, Mason L, Alexander KT, Odhiambo FO, Eleveld A, Mohammed A, van Eijk AM, Edwards RT, Vulule J, Faragher B, Laserson KF (2016). Menstrual cups and sanitary pads to reduce school attrition, and sexually transmitted and reproductive tract infections: a cluster randomised controlled feasibility study in rural western Kenya. BMJ Open.

[31] Baker KK, Padhi B, Torondel B, Das P, Dutta A, Sahoo KC, Das B, Dreibelbis R, Caruso B, Freeman MC, Sager L (2017). From menarche to menopause: a population-based assessment of water, sanitation, and hygiene risk factors for reproductive tract infection symptoms over life stages in rural girls and women in India. PLoS One.

[32] Garg R, Goyal S, Gupta S (2012). India moves towards menstrual hygiene: subsidized sanitary napkins for rural adolescent girls issues and challenges. Matern Child Health J.

[33] Sommer M, Hirsch JS, Nathanson C, Parker RG (2015). Comfortably, safely, and without shame: defining menstrual hygiene management as a public health issue. Am J Public Health.

[34] Ness RB, Kip KE, Soper DE, Stamm CA, Rice P, Richter HE (2006). Variability of bacterial vaginosis over 6- to 12-month intervals. Sex Transm Dis.

[35] Fethers KA, Fairley CK, Morton A, Hocking JS, Hopkins C, Kennedy LJ, Fehler G, Bradshaw CS (2009). Early sexual experiences and risk factors for bacterial vaginosis. J Infect Dis.

[36] Witkin SS, Linhares IM, Giraldo P, Ledger WJ (2007). An altered immunity hypothesis for the development of symptomatic bacterial vaginosis. Clin Infect Dis.

[37] Aila NAE, Tency I, Claeys G, Verstraelen H, Saerens B, Santiago GLS, Backer ED, Cools P, Temmerman M, Verhelst R, Vaneechoutte M (2009). Identification and genotyping of bacteria from paired vaginal and rectal samples from pregnant women indicates similarity between vaginal and rectal microflora. BMC Infect Dis.

[38] Mathiyalagen P, Peramasamy B, Vasudevan K, Basu M, Cherian J, Sundar B (2017). A descriptive cross-sectional study on menstrual hygiene and perceived reproductive morbidity among adolescent girls in a union territory, India. J Family Med Prim Care.

[39] Preethi V, Mandal J, Halder A, Parija SC (2011). Trichomoniasis: An update. Trop Parasitol..

[40] Sommer M, Caruso BA, Sahin M, Calderon T, Cavill S, Mahon T, Phillips-Howard PA (2016). A time for global action: addressing girls’ menstrual hygiene management needs in schools. PLoS Med.

[41] Montgomery P, Ryus CR, Dolan CS, Dopson S, Scott LM (2012). Sanitary pad interventions for girls' education in Ghana: a pilot study. PLoS One.

[42] Crofts T, Fisher J (2012). Menstrual hygiene in Ugandan schools: an investigation of low-cost sanitary pads. J Water Sanit Hyg Dev..

[43] Garg R, Goyal S, Gupta S (2012). India moves towards menstrual hygiene: subsidized sanitary napkins for rural adolescent girls—issues and challenges. Matern Child Health J.

[44] Sommer M, Kjellén M, Pensulo C (2013). Girls' and women's unmet needs for menstrual hygiene management (MHM): The interactions between MHM and sanitation systems in low-income countries. J Water Sanit Hyg Dev.

[45] Kengne P, Veas F, Vidal N, Rey JL, Cuny G (1994). Trichomonas vagnialis: repeated DNA target for highly sensitive and specific polymerase chain reaction diagnosis. Cell Mol Biol.

[46] Simpson P, Higgins G, Qiao M, Waddell R, Kok T (2007). Real-time PCRs for detection of trichomonas vaginalis b-tubulin and 18S rRNA genes in female genital specimens. J Med Microbiol.

