# Supplementary material for: Association between unhygienic menstrual management practices and prevalence of lower reproductive tract infections: a hospital-based cross-sectional study in Odisha, India
Source: BMC Infect Dis. 2018 Sep 21;18:473. doi: 10.1186/s12879-018-3384-2 (PMC6150969; doi:10.1186/s12879-018-3384-2)
Supplement: Supplementary file 1 — Variable table with Definitions (Table that present all the variables measured in the study and their definitions). (DOC 62 kb) [file 12879_2018_3384_MOESM1_ESM.doc]

**Supplementary File 1: Variable table with Definitions.**

| **Variable** | **Level** | **Definition** |
| --- | --- | --- |
| Socio-economic confounders | | |
| Age | 18-25 years |  |
|  | 26-35 years |  |
|  | 36-45 years |  |
| Marital status | Never married | Single marital status |
|  | Married | Married |
|  | Divorcee & Widow | Divorced/widowed |
| Religion | Hindu |  |
|  | Muslim |  |
|  | Christian |  |
| Caste/Tribe | SC | Schedule Caste |
|  | ST | Schedule Tribe |
|  | OBC | Other Backward Class |
|  | Other Caste |  |
| Occupation | Employed |  |
|  | Housewife |  |
|  | Student |  |
|  | Other |  |
| Education | None | No formal education |
|  | 5^th^ – 10^th^ grade | Between 5^th^ – 10^th^ grade |
|  | 12^th^ grade or higher | 12^th^ grade or higher |
| Family members in household | 3 or less |  |
|  | 4-5 |  |
|  | More than 5 |  |
| Monthly income | <5,000.00 |  |
|  | 5,000.00-10,000.00 |  |
|  | >10,000.00 |  |
|  | Don’t know |  |
| Exposures of Interest | | |
| Sanitation access | Yes | Having a latrine at home |
|  | No | Not having a latrine at home |
| Place where water source is located | In the house | Inside the house |
|  | In the yard | At the yard of the own house |
|  | At relatives/  Neighbour house or yard | At a relative place or a neighbor house/yard |
|  | At a public location | At a public space |
| Absorbent Materials | Disposable | Disposable sanitary pads |
|  | Reusable | Reusable cloths/towels |
| Type of reusable material use | Old cotton fabric |  |
|  | Old silk/nylon fabric |  |
| Frequency of changing absorbent | Once a day |  |
|  | Twice a day |  |
|  | Three times a day or more |  |
| Place of changing absorbent | In a toilet facility | Inside toilet facility |
|  | Outside a toilet facility | Outside toilet facility |
| Place to wash absorbent | Inside toilet stall |  |
|  | At tube well or yard |  |
| How do you dry the cloth | Dry it in sun/open space |  |
|  | Dry it inside the house |  |
| Storage of cloth | Polythene | Wrapped in polythene |
|  | Another material | Wrapped in another material |
| Storage of cloth for next use | Cupboard | Within the cupboard in the changing room |
|  | In the Toilet | Hidden inside the toilet compartment |
| Type of washing during menstruation | Only vaginal wash |  |
|  | Bath of full body |  |
| Frequency of washing during menstruation | Twice or more per day |  |
|  | Once a day |  |
