# Supplementary material for: Association between unhygienic menstrual management practices and prevalence of lower reproductive tract infections: a hospital-based cross-sectional study in Odisha, India
Source: BMC Infect Dis. 2018 Sep 21;18:473. doi: 10.1186/s12879-018-3384-2 (PMC6150969; doi:10.1186/s12879-018-3384-2)
Supplement: Supplementary file 2 — Diagnosis of BV, TV, and Candida infection. Detail explanation about laboratory methods used to diagnosed the studied infections [45, 46]. (DOCX 12 kb) [file 12879_2018_3384_MOESM2_ESM.docx]

**Supplementary file 2**:

**Diagnosis of BV, TV, and Candida infection.**

BV was diagnosed using Nugent’s laboratory diagnostic criteria [23]. In brief, slides prepared from the vaginal swabs were stained with Gram’s stain and examined for specific bacterial morphotypes. A Nugent score (NS) between 0-10 was generated using the Nugent criteria. A NS of 0 to 3 was interpreted as normal or negative for BV, a score of 4 to 10 as intermediate/abnormal for BV or positive for BV.

Presence of TV was identified by nucleic acid amplification tests. DNA was extracted from stored swabs by phenol-chloroform method [24]. Diagnostic PCR was used to detect TV infection. Two primer sets were used: TVK3/TVK7(5’-ATTGTCGAACATTGGTCTTACCCTC-3’)/(5’-TCTGTGCCGTCTTCAAGTATGC-3’) [45] and TV16f-2/TV16r-2 (5’-TGAATCAACACGGGGAAAC-3’)/(5’-ACCCTCTAAGGCTCGCAGT-3’) [46]. All samples were considered positive if TV was detected by PCR with either of the two primers. For Quality Assurance/Quality Control (QA/QC) we ran a positive control (kindly supplied by Prof. Jane Carlton, New York University, USA) and a negative control with each batch.

AlbiQuick^TM^ rapid test (HARDY Diagnostic, CA, USA) was used for identification of *Candida albicans* as per manufacturer’s instructions. Briefly, the vaginal swab was cultured in Blood Agar and Chocolate Agar plate for 48 hr at 37ºC. After 48 hr, a sample was taken in a sterile loop and smearedon the test card for 5 min at room temperature. A bright blue-white or blue-purple fluorescence was observed for positive *Candida albicans* infection and no fluorescence was seen for negative samples.
